# Targeting PDGF Signaling in Carcinoma-Associated Fibroblasts Controls Cervical Cancer in Mouse Model

**DOI:** 10.1371/journal.pmed.0050024

**Published:** 2008-01-29

**Authors:** Rakesh K Jain, Johanna Lahdenranta, Dai Fukumura

## Abstract

The authors discuss a new study of a paracrine regulatory circuit centered on PDGF receptor signaling in cancer-associated fibroblasts and pericytes of a mouse model of cervical carcinoma.

Cervical cancer is one of the most prevalent malignancies in women worldwide and is the leading cause of cancer death for women in developing countries [1]. While early detection via the Pap test as well as treatment by surgery and chemoradiotherapy has reduced mortality from this disease, the prognosis is poor if the disease is detected at an advanced stage [2]. Thus new treatment strategies for cervical cancer are needed.

In this issue of *PLoS Medicine*, Kristian Pietras and colleagues, using a mouse model of cervical carcinogenesis, provide compelling evidence that targeting platelet-derived growth factor (PDGF) signaling, primarily in carcinoma-associated fibroblasts (CAFs), can slow the progression of this disease and even impair the growth of invasive carcinomas [3]. By offering preliminary evidence for the presence of PDGF receptors in a limited number of human cervical cancer biopsies, these authors also suggest that the drugs approved by the United States Food and Drug Administration (FDA) that target PDGF signaling, such as imatinib mesylate (Gleevec in the US; Glivec in Europe and Australia, Novartis), be tested in the clinic for this malignancy.

## CAFs as Key Players in Cervical Carcinogenesis

The present study builds on previous work by this group and others where targeting PDGF signaling has been based primarily on the presence of PDGF receptors on the pericytes and endothelial cells of tumors. Targeting PDGF receptors on tumor pericytes can destabilize tumor blood vessels, making them more vulnerable to anti-vascular endothelial growth factor (VEGF) therapies [4]. Targeting PDGF receptors on the tumor's endothelial cells can have direct anti-vascular effects [5]. In the new study, Pietras and colleagues offer evidence that targeting PDGF signaling in the carcinoma-associated fibroblasts plays a central role in the tumor response to PDGF receptor (PDGFR) blockade.

**Linked Research Article**

This Perspective discusses the following new study published in *PLoS Medicine:*

Pietras K, Pahler J, Bergers G, Hanahan D (2008) Functions of paracrine PDGF signaling in the proangiogenic tumor stroma revealed by pharmacological targeting. PLoS Med 5(1): e19. doi:10.1371/journal.pmed.0050019

Douglas Hanahan and colleagues investigate a paracrine regulatory circuit centered upon PDGF receptor signaling in cancer-associated fibroblasts and pericytes of a mouse model of cervical carcinogenesis.

Using a previously developed model of cervical carcinogenesis, these authors show that PDGF receptors are primarily present in CAFs (PDGFRa and ß) and pericytes (PDGFRß). In contrast, PDGF ligands (most abundantly PDGF-C) are present almost exclusively in cancer cells. They further show that blocking PDGF receptors a and ß signaling in the CAFs—using either imatinib (a tyrosine kinase inhibitor) or monoclonal antibodies against these two receptors—can repress expression of fibroblast growth factor (FGF)-2, a potent pro-angiogenic molecule. This in turn can block angiogenesis in these tumors in which endothelial cells express fibroblast growth factor receptor 1 (FGFR1). They also show that these agents reduce pericyte coverage on tumor blood vessels—as reported previously by a number of laboratories [4,6,7]. Finally, they show that imatinib decreases expression of FGF-7 (keratinocyte growth factor) in CAFs, and propose that this could potentially inhibit growth of FGFR-expressing cervical cancer cells. Putting this evidence together, these authors offer a potential approach to treat cervical cancer by targeting PDGF paracrine signaling between stromal and epithelial cells using imatinib.

## The Complexity of Targeting PDGF Signaling

Originally developed as a bcr-abl kinase inhibitor, imatinib has served as a poster child for targeted therapy. Based on its dramatic effects on chronic myelogenous leukemia (CML), it was approved in 2001 by the FDA for CML and hailed as a “magic bullet” [8]. Later it was shown to be effective against gastrointestinal stromal tumors because of its activity against c-kit and PDGFRa expressed in cancer cells in this disease [9]. However, as a single agent, imatinib has not yet proven efficacious in PDGFR-expressing common solid tumors in any phase II clinical trials. Furthermore, although a small fraction of patients with glioblastoma multiforme (GBM) responded to imatinib, there was no correlation between patient survival and the tumor cell expression of the molecular targets of imatinib [10]. PDGFRs are also expressed on the vascular endothelium of glioblastomas [11]. Thus it is possible that imatinib might have led to a direct anti-vascular effect on these vessels in GBMs in addition to the indirect effect through FGF2 downregulation demonstrated in Pietras and colleagues' study.

Despite these exciting results, several important issues remain to be solved. The potential benefits of targeting PDGFRs in the tumor stromal cells must be balanced by the adverse effects of imatinib on normal tissues, such as fluid retention, pleural effusions, and ascites formation [12,13]. Furthermore, the loss of pericytes in tumor vessels that results from blocking PDGFRs can further destabilize tumor vessels and make them more abnormal. This abnormality, in turn, can impair blood flow, cause poor drug delivery when other agents are given concomitantly, and create a hostile microenvironment, characterized by hypoxia and acidosis, which makes tumors more aggressive and resistant to many treatments [14]. Lack of pericyte coverage may also facilitate tumor cell metastasis [15]. Finally, the phenotype of stromal fibroblasts is known to vary from one organ to the next. Thus, blocking PDGF signaling in CAFs might slow the growth of a tumor in its primary site, but might not have a similar effect on tumor metastases in secondary sites.

These considerations underscore the complexity of targeting paracrine PDGF signaling between stromal and neoplastic epithelial cells. These limitations not withstanding, Pietras and colleagues' study demonstrates how targeted alteration of CAF functions can lead to both anti-tumorigenic and anti-angiogenic effects, improving the disease outcome. These results offer a foundation for development of new approaches to the treatment of cervical cancer where suppression of PDGF-induced CAF functions in combination with conventional cytotoxic therapy and/or VEGF-dependent anti-angiogenic therapy might prove beneficial.

## Abbreviations

CAF: carcinoma-associated fibroblast
CML: chronic myelogenous leukemia
FDA: Food and Drug Administration
FGF: fibroblast growth factor
FGFR: fibroblast growth factor receptor
GBM: glioblastoma multiforme
PDGF: platelet-derived growth factor
PDGFR: platelet-derived growth factor receptor
VEGF: vascular endothelial growth factor
